# Diagnostic challenges and management of choledochal cyst in an 11-year-old child: a delayed diagnosis (a case report)

**DOI:** 10.11604/pamj.2021.40.224.32549

**Published:** 2021-12-15

**Authors:** Novath Julius Ngowi, Kaitila Murusuri, Ally Mwanga, Yona Ringo

**Affiliations:** 1 Department of Surgery, Muhimbili University of Health and Allied Sciences, Dar es Salaam, Tanzania,; 2 Department of Surgery, Muhimbili National Hospital, Dar es Salaam, Tanzania

**Keywords:** Choledochal cyst, cholangioenterostomy, hepatojejunostomy, case report

## Abstract

Choledochal cyst are rare congenital disease of the biliary tree. It presenting as cystic dilatations of the biliary tree can involve the extrahepatic biliary radicles, the intrahepatic biliary radicles or both. They are typically a surgical problem of infancy and childhood, but less than a quarter of the patients the diagnosis is delayed until adulthood as it presenting with vague and nonspecific signs and symptoms. In a case with biliary symptoms, abdominal ultrasound scan is the initial imaging modality of choice. Precise and accurate delineation of the biliary system mandates cholangiography with the advantage of non-invasive magnetic resonance cholangiopancreatography (MRCP) over endoscopic retrograde cholangiopancreatography. A case report of 11-year-old Tanzania girl with abdominal swelling and jaundice presented to a health facility for evaluation. She complained of abdominal swelling that her mother noticed when she was 3 years old, located above the umbilicus and since then it was not changed its size until 8 years later when it rapidly increased in size associated with non-specific dull pain. Abdominal computed tomography (CT) scan was done showed A well-defined hypo-attenuated non-enhancing retro-gastric cyst. Percutaneous transhepatic cholangiopancreatography (PTC) was performed due to inconclusive findings from CT-scan showed extrahepatic huge cystic dilatation, dilated central right hepatic ducts, left intrahepatic ducts failed to be visualize. Diagnosis of choledochal cyst type isovaleric acidaemia (IVA) was made. Explorative laparotomy was done, huge cystic mass occupying common bile duct was seen below the liver with distended gallbladder covered with visceral peritoneum. Second part of duodenum, pancreases and transverse colon was adhered to the inferior surface of the mass that further make difficult for cyst excision and reconstruction. Cyst was decongested and cholangioenterostomy with Roue-en-Y reconstruction was made. Cholecystectomy was done, hemostasis archived abdomen closed and patient sent to Intensive care units (ICU). Despite of advanced diagnostic modalities, delayed diagnosis of choledochal cyst can be a challenge due to its vague and nonspecific signs and symptoms. Excision of the cyst and reconstruction by hepatojejunostomy as the standard therapy could be difficult due to its biliary complications such as adhesion and infection and hence cyst-enterostomy drainage procedure can be done as option for relief of patient discomfort and prevent further complications.

## Introduction

Choledochal cysts are rare congenital bile duct anomalies, presenting as cystic dilatations of the biliary tree can involve the extrahepatic biliary radicles, the intrahepatic biliary radicles, or both. They may occur as a single cyst or in multiples within the biliary tree. They are typically a surgical problem of infancy and childhood, but less than a quarter of the patients the diagnosis is delayed until adulthood. The presentation is often vague and nonspecific. The so-called classic triad of intermittent jaundice, abdominal mass, and pain is found in a minority of cases according to most case series [1]. The most frequently seen presentation is abdominal pain which is a nonspecific symptom and usually associated with a relatively late diagnosis followed by jaundice as it usually associated with early diagnosis. In a case with biliary symptoms, abdominal ultrasound scan is the initial imaging modality of choice. Precise and accurate delineation of the biliary system mandates cholangiography with the advantage of non-invasive MRCP over endoscopic retrograde cholangiopancreatography [2]. Choledochal cysts present differently in adults and children; whereas children present with the classic triad, adults present with common biliary or infective complication. This case report highlights the difficulties involved in making a correct diagnosis and the operative treatment for a choledochal cyst.

## Patient and observation

**Patient information:** an 11-year-old Tanzania girl with abdominal swelling and jaundice presented to a health facility for evaluation. She complained of abdominal swelling that her mother noticed when she was 3 years old, located above the umbilicus and since then it was not changed its size until 8 years later when it rapidly increased in size associated with non-specific dull pain. During rapid increase in size of the swelling she started presenting with intermittent yellowish discoloration of eyes. During the course of the illness, she had no history of vomiting, difficult in passing stool or passing bloody stool or black tarry stool. She reported history of passing deep colored urine during the period of yellowish discoloration with no history skin itching or passing clay colored stool. The patient had received treatment from multiple primary health facilities consisting of antibiotics and analgesics due to the complaints without relief until when she was referred to Tertiary Hospital.

**Clinical findings:** the child presented in hospital fully conscious and ambulant, some pallor on her conjunctiva and tinge jaundice. She had no enlarged periphery lympnodes, no lower limb oedema and vital signs were normal. Her body mass index (BMI) was 17kg/m^3^ indicated that she was underweight. Abdomen was symmetrically distended with girth of 74cm at its largest point of circumference, moves with respiration with visible superficial veins. Mass was palpable on right hypochondriac region extending to involve right lumbar, right iliac, umbilical regions with smooth margin and edges, firm, tender on deep palpation, unable to go above it but its lower margin and edges were felt. Its lower edge was measured to be 18cm from right subcostal margin. Liver span was 20cm. It was generalized dull percussion note and normal bowel sound heard.

**Figure 1:**
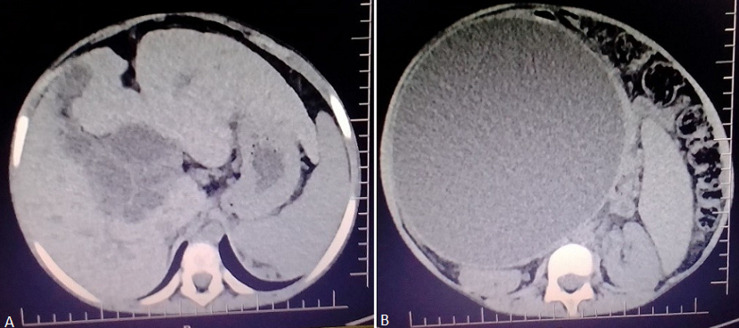
A, B) triphasic CT scan of the abdomen showed a well-defined retro-gastric cyst with dilatation of intra and extra biliary tree; differential diagnosis of choledochal cyst and pancreatic pseudocyst was made

The clinical laboratory was done that revealed elevated markers of liver damage alanine aminotransferase and aspartate transaminase (ALT and AST), elevated markers for the biliary obstruction gamma-glutamyltransferase and alanine aminotransferase (GGT and ALT), serum bilirubin level was elevated with more than half being conjugated bilirubin level. She had mild elevated bleeding indices and microcytic hypochromic anemia. The patient stayed in the ward for one month while the investigations were being done and received vitamin K injection, transfused two units of blood and she was given analgesics for the abdominal pain. Abdominal CT scan was done showed A well-defined hypo-attenuated non-enhancing retro-gastric cyst is noted measuring approximately (17.5*15.8*16.3) cm^3^ with no septations. It has a thin wall, no calcification, no solid component. There is a dilatation of intra and extra biliary tree. The liver is normal in size, shape and density. Spleen and Vascular structure appeared normal (Figure 1A, B).

**Figure 2:**
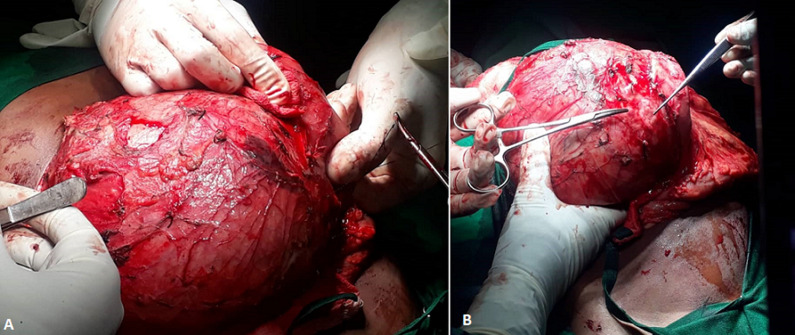
C, D) huge cystic mass was seen, arising from the liver extending to the duodenum, pancreas, duodenum and transverse colon adhered to the mass

Percutaneous transhepatic cholangiopancreatography (PTC) was performed because the computer tomography was considered inconclusive for definitive surgical management as revealed retro-gastric cyst with differential diagnosis of choledochal cyst and pancreatic cyst. During PTC with assistance with ultrasonography, contrast injected into Gall bladder, it was seen flowing through the cystic duct and emptying into the extrahepatic huge cystic dilatation. Right intrahepatic duct was assessed, contrast was injected showed cystically dilated central right hepatic ducts, left intrahepatic ducts failed to be visualize. Diagnosis of choledochal cyst type IVA was made. Panel discussion was made with pediatric surgeons and surgical gastroenterologist, the image findings were reviewed during the discussion and conclusion for explorative laparotomy with possible cyst-enteric drainage procedure was made. Laparotomy was done, through upper abdominal transverse incision abdomen was opened in layers with aseptic techniques, huge cystic mass was seen, arising from common bile duct below the liver (Figure 2A, B).

Pancreas, duodenum and transverse colon adhered below the mass. Liver was hard and nodulated with rough surface. The mobilization of transverse colon from the cyst was done. Duodenum and pancreas were not mobilized due to the risk of damage to pancreatic duct. Cholecystectomy was done. Cyst was decongested and drained 2.4 liters of bile and sent for biochemistry analysis. Retro-colic cholangioenterostomy with Roue-en-Y reconstruction was made. Biopsy was taken from liver, cyst wall and gall bladder for histopathology. Abdominal lavage with warm normal saline was done, and abdomen was closed in layers. During the operation the patient received two units of blood and single unit of fresh frozen plasma, the she was sent to ICU. The patient was full recovered from ICU then sent back to the ward and discharged after two weeks continue to be followed up in the clinic.

**Informed consent:** written informed consent was obtained from the patient's parents for publication of this case report and any accompanying images. A copy of the written consent is available for review.

## Discussion

Choledochal cyst are rare congenital disease of the biliary tree. It presenting with vague and nonspecific features usually associated with a relatively late diagnosis. These presentations include upper abdominal pain and jaundice which are common in many other illnesses of the upper gastrointestinal tract. The classical triad of jaundice, right upper quadrant mass, and abdominal pain is present in only a minority of patients (0-17%) [3]. Similar to this case, a 11 years old child presented with non-progressive abdominal swelling while she was 3 years-old for 8 years with non-specific abdominal pain and no other gastrointestinal symptoms. Other presenting features of choledochal cysts are cholangitis, pancreatitis, and biliary peritonitis from cyst rupture that the child did not have that also contributed to the delayed on seeking medical attention.

Abdominal ultrasonography remains the initial imaging modality of choice in gastrointestinal tract disease presenting with biliary symptoms as it is sensitive in the detection of cystic structures but not specific in delineate the structural origin. Computed tomography (CT) is infrequently required in the situation where the distal common bile duct is not visualized due to bowel gas. Ultrasound and CT are excellent modalities for detecting cystic right upper abdominal lesions and for assessing their size and extent, but the biliary origin of the cyst may not be always reliably commented [4]. In this case the diagnosis was missed initial while the she was admitted inconclusive from the abdominal sonography done at local health facility and CT scan done at tertiary hospital as it failed to detect the biliary origin of the cyst.

Magnetic resonance cholangiopancreatography is emerging as a highly sensitive, safe, and noninvasive diagnostic preoperative technique for the detection of choledochal cysts although has limited capacity to detect associated ductal anomalies or small choledochocele [5]. In this case report, MRCP was not priority as the differential for choledochal cyst was missed before from the CT scan imaging done while patient was admitted in hospital.

The treatment of a choledochal cyst has changed. In the past, a cysto-jejunostomy was the standard procedure. Currently, excision of the cyst and reconstruction by hepatojejunostomy is the standard therapy [6]. This this case report demonstrates the intraoperative difficulties in mobilizing the structures adhered to the cyst, the further separation of the structures from cyst would increase chance of iatrogenic damage to pancreas and duodenum. This intraoperative exploration of the cyst should be performed and prompt to dispute the preoperative diagnosis and define the definitive surgical management compared to the inconclusive result obtained initially from CT scan imaging.

## Conclusion

Choledochal cyst are rare congenital disease during childhood presenting with nonspecific sign and symptoms which associate with relative late diagnosis. Excision of the cyst and reconstruction by hepatojejunostomy as the standard therapy could be challenge due to its biliary or infection complication and hence cystenterostomy drainage procedure can be done as option for relief of patient discomfort and prevent further complications.
